# A novel risk model based on anoikis: Predicting prognosis and immune infiltration in cutaneous melanoma

**DOI:** 10.3389/fphar.2022.1090857

**Published:** 2023-01-16

**Authors:** Yi Zhou, Chen Wang, Yifang Chen, Wei Zhang, Zailin Fu, Jianbo Li, Jie Zheng, Minghua Xie

**Affiliations:** ^1^ Department of Pharmacy, First People’s Hospital of Linping District, Hangzhou, Zhejiang, China; ^2^ The Cancer Hospital of the University of Chinese Academy of Sciences (Zhejiang Cancer Hospital), Institute of Basic Medicine and Cancer (IBMC), Chinese Academy of Sciences, Hangzhou, China; ^3^ School of Medicine, Chongqing University, Chongqing, China

**Keywords:** cutaneous melanoma, anoikis-related genes, risk model, immune microenvironment, prognosis

## Abstract

Cutaneous melanoma (CM) is a highly aggressive malignancy with a dimal prognosis and limited treatment options. Anoikis is believed to involve in the regeneration, migration, and metastasis of tumor. The exact role of anoikis-related genes (ARGs) in the development and progression of cutaneous melanoma, however, remains elusive. Four ARGs (*SNAI2*, *TFDP1*, *IKBKG*, and *MCL1*) with significant differential expression were selected through Cox regression and LASSO analyses. Data for internal and external cohorts validated the accuracy and clinical utility of the prognostic risk model based on ARGs. The Kaplan–Meier curve indicated a much better overall survival rate of low-risk patients. Notably, we also found that the action of ARGs in the CM was mediated by immune-related signaling pathways. Consensus clustering and TIME landscape analysis also indicated that the low-risk score patients have excellent immune status. Moreover, the results of immunotherapy response and drug sensitivity also confirmed the potential implications of informing individualized immune therapeutic strategies for CM. Collectively, the predictive risk model constructed based on ARGs provides an excellent and accurate prediction tool for CM patients. This present research provides a rationale for the joint application of targeted therapy and immunotherapy in CM treatment. The approach could have great therapeutic value and make a contribution to personalized medicine therapy.

## Introduction

Cutaneous melanoma (CM) is a devastatingly aggressive malignancy with increasing incidence and poor prognosis (Dika et al., 2019; Yu et al., 2022). The estimated annual incidence is approximatelymore than 100,000 cases globally and the mortality of CM accounts for 80%–85% of all skin cancer related death (Yang et al., 2022). CM is notorious for its metastasis and loco-regional recurrence, which always results in a poor prognosis and high mortality for patients. Early diagnosis and prompt surgical removal are reliable treatments for localized CM; however, this strategy does not benefit patients with metastatic melanoma of the later stages (Antohe et al., 2022). With development of immunotherapies and targeted therapies, treatment options for CM have increased dramatically. Remarkably, an almost inevitable acquired resistance to therapy is another hallmark of CM (Swami et al., 2020; Gullo et al., 2022). Thus, in-depth understanding the molecular mechanism of CM development is imperative to identify novel diagnostic and therapeutic biomarkers for CM patients.

Anoikis, as a programmed cell death, is a potential barrier to cancer cell metastasis. Previous studies have pointed that the epithelial or endothelial cells detached from extracellular matrix (ECM) succumb to classical apoptosis commonly known as anoikis (Chi et al., 2022; Sun et al., 2022). Evasion from apoptosis is one of the essential changes in the malignant transformation of cells. In contrast with healthy cells, tumor cells can evade anoikis, which contributes to tumor progression and metastasis (de Sousa Mesquita et al., 2017; Kakavandi et al., 2018). During tumorigenesis, the isolated tumor cells bypass death signaling pathways and escape immune recognition, which is the main reason for the occurrence of anoikis resistance (Shi et al., 2022). Numerous studies have highlighted the important role of anoikis resistance in tumor migration and metastasis (Saharat et al., 2018; Fanfone et al., 2022). Thus, to better understand the progression, metastasis and chemoresistance of CM, it is necessary to recognize the functioning of anoikis.

The tumor microenvironment (TME) is indispensable in the tumor development and progression (Kyriakou and Melachrinou, 2020). Specifically, the tumor immune microenvironment (TIME) has drawn much attention as a main contributor of CM progression and metastasis, apoptosis, and invasion (Gray et al., 2017; Virtuoso et al., 2022). Wang et al. pointed out that TIME was a potential biomarker for CM cancer immunotherapy (Wang et al., 2020). Several lines of evidence have highlighted that immunotherapies that block immune checkpoints, such as programmed cell death 1 (*PD-1*)/*PD-1* ligand (*PD-L1*) axes and cytotoxic T lymphocyte antigen-4 (*CTLA-4/CD28*), were related to the prognosis of CM patients (Herbreteau et al., 2018; Patel et al., 2022). Of note, a significant number of patients experience drug resistance and metastasis and even died due to treatment-related adverse drug reactions. Intriguingly, anoikis is an important barrier to metastasis. Gaining anoikis resistance is a prerequisite for tumor migration and metastasis of CM. Guadamillas et al. (2011) showed that the abnormal microenvironment also helps the cancer evade anoikis. Yet, to date, systematic research aimed at anoikis-related genes (ARGs) and TIME in CM is still lacking.

Accordingly, the present study mainly focused on the association between ARGs and the clinicopathological characteristics of CM based on the analysis of The Cancer Genome Atlas database (TCGA) and the Gene Expression Omnibus (GEO) database. A novel risk model was constructed based on four prognostic ARGs. Next, the correlation of risk score and the immune microenvironment landscape of patients with CM was comprehensively explored. This research is expected to help in the conception of new perspectives for the design of potential therapeutic strategies and antitumor targets for CM.

## Materials and methods

### Data collection

The transcriptome matrix and clinical information were gained from TCGA (https://portal.gdc.cancer.gov/) and GEO (https://www.ncbi.nlm.nih.gov/geo/). GSE65904 was downloaded from the GEO and annotated based on the microarray platform GPL10558.210. CM samples were obtained from the GEO. The R package “sva” was utilized to remove the batch effect of the transcriptome matrix in the TCGA and GEO databases. The M stage (M0 vs. M1) of these cancers was excluded in this study.

### ARG collection and risk model construction

Thirty-four ARGs were derived from the MSigDB database (https://www.gsea-msigdb.org/gsea/) (Supplementary Table S1). The prognostic ARGs associated with the overall survival (OS) rate were identified by univariate Cox regression. The LASSO algorithm was used to choose the characteristic prognostic ARGs by applying the R package “glmnet.” According to the coefficients calculated by multivariate Cox regression, the risk score of each sample was calculated by the following formula: risk score = coeff * expression of ARGs. The patients with CM were categorized into low- and high-risk groups based on the median risk score. The OS rate of CM patients was evaluated by the Kaplan–Meier survival curve *via* a log-rank algorithm using the R package “survival.”

### ARG prognostic signatures validation

To validate the independence of the ARG prognostic signatures, the TCGA cohort was used as the internal cohort, and the GEO cohort was used as the external cohort. With the ratio set at 1:1, the CM samples were randomly divided into a training cohort and a test cohort *via* R package “caret.” Meanwhile, GSE65904 was used to validate the stability of the ARG prognostic signatures as an external validation cohort.

### Consensus clustering analysis

Consensus cluster analysis was utilized to classify the CM samples into different molecular subtypes *via* the R package “Consensus Cluster Plus.” The CM patients were randomized into two subtypes in this study based on the consensus matrix (K = 2–9). For the clustering analysis, patients were clustered based on the grounds of partitioning around medoids with “Euclidean” distances, and 1,000 verifications were performed.

### ARGs prognostic independence evaluation

Cox analyses were performed to evaluate that the risk model of ARG prognostic signatures was a clinical independent factor for CM. The R package “survival” was used to evaluate the OS rate of CM patients with different clinicopathological characteristics. The R package “rms” was used to construct a nomogram to evaluate the OS rate of patients with CM in 1, 3, and 5 years. A calibration diagram was used to assess the accuracy of the nomograms. The capability of the risk model in evaluating prognosis for CM was validated using the “timeROC” R package.

### Functional enrichment analysis of differential expression genes (DEGs)

The DEGs in the different risk groups were identified through the R package “limma.” |Fold change| ≥ 2 and *p* < .05 were set as the threshold to select DEGs and visualized in a volcano diagram *via* the R package “ggplot2.” The gene set variation analysis (GSVA) algorithm was used to calculate the activity of the Kyoto Encyclopedia of Genes and Genomes (KEGG) terms of the CM patients *via* the R package “GSVA.” Gene oncology (GO) and KEGG enrichment analysis were determined *via* the clusterProfiler package (Yu et al., 2012).

### Analysis of immune infiltration landscape

The R package “estimate” was used to evaluate the stromal and immune cells of the CM samples, include tumor purity, stromal, ESTIMATE, and immune scores. The proportion of 22-type immune cells was evaluated using the CIBERSORT algorithm *via* the “CIBERSORT R script v1.03” script. The 23-type immune cells were evaluated by the ssGSEA algorithm. In addition, the immune function score of the CM samples was estimated by the R package “GSVA”, and 14 immune function scores were evaluated. The R package “ggplot2” was utilized to investigate the correlation of prognostic ARGs and immune infiltration cells *via* the Spearman-ranked correlation algorithm.

### Immunotherapy response and drug sensitivity analysis

The immunophenoscore (IPS) database, tumor immune dysfunction and exclusion (TIDE) scores, and an anti-*PD1*/*PD-L1* immunotherapy cohort (IMvigor210) were utilized to evaluate the response of immunotherapy for CM patients. The result of IPS was collected from the TCIA database (https://tcia.at/home). The response of anti-*PD1*/*PD-L1* for CM was evaluated by the Imvigor210 cohort. In addition, the TIDE score of CM was evaluated *via* the TIDE database (http://tide.dfci.harvard.edu). The drug sensitivity (IC50) of each CM sample was predicted by “pRRophetic” according to the GDSC database. The Spearman-ranked correlation algorithm was used to analyze the relationship between the risk score and drug sensitivity (IC50).

### Statistical analysis

All statistical analyses were carried out in the R software (version 4.1.0) (https://cran.r-project.org/) and Perl software. The Spearman-ranked correlation algorithm was used to estimate the correlations between different data points, and a *p*-value <.05 was regarded as statistically significant. The Wilcoxon rank-sum test assessed the significance between the two groups, and *p* < .05 was judged to be statistically significant.

## Result

### Risk model construction based on the ARG prognostic signatures

**FIGURE 1:**
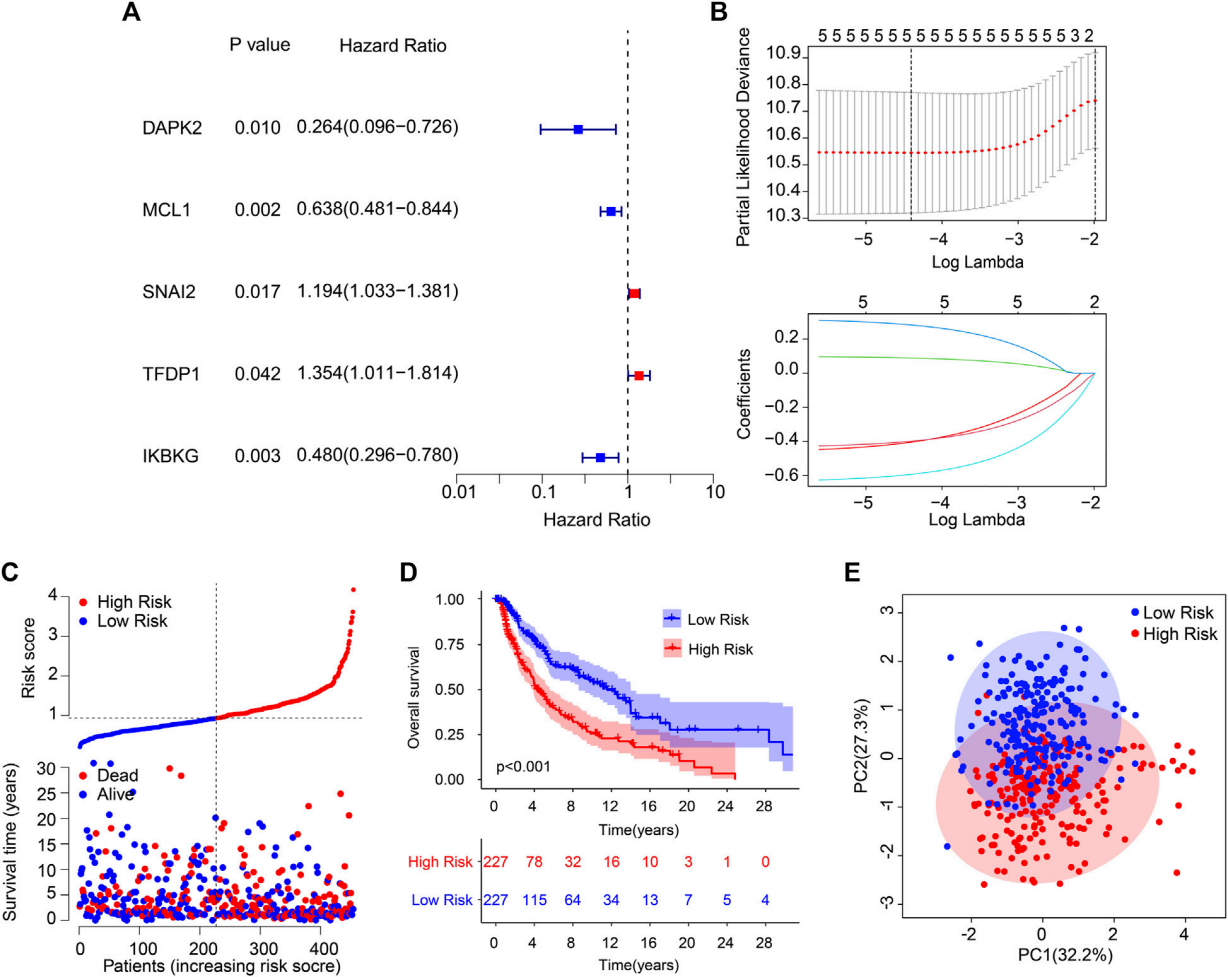
Risk model construction based on the prognostic ARGs in CM. **(A)** Identification of prognostic ARGs *via* the univariate Cox regression analysis. **(B)** The result of LASSO regression analysis. **(C)** The Kaplan–Meier survival curve indicates that the low-risk score patients have higher OS rates. **(D)** The classification of the CM patients according to the ARG prognostic signatures and the scatter dot plot reveals the correlation between the ARG prognostic signatures and survival time. **(E)** A clear distribution between the different risk groups is revealed by principal component analysis.

A new prognostic risk model based on ARGs was constructed to assess the prognostic value of the ARGs in patients with CM. As represented in Figures 1A, B, five prognostic ARGs associated with the CM OS rate were verified by the LASSO analysis. Following the multivariate Cox regression analyses, four independent prognostic ARGs that evaluate the prognosis of CM were validated to construct the risk model, including *SNAI2*, *TEDP1*, *IKBKG*, and *MCL1*. The Kaplan–Meier survival curve showed that the OS rate in the patients with high-risk scores was relatively lower (Figures 1C, D). Furthermore, a remarkable distinction was observed between the low- and high-risk groups *via* the principal component analysis (Figure 1E).

### The prediction risk model combining the prognostic signature of ARGs evaluates the CM prognosis

**FIGURE 2:**
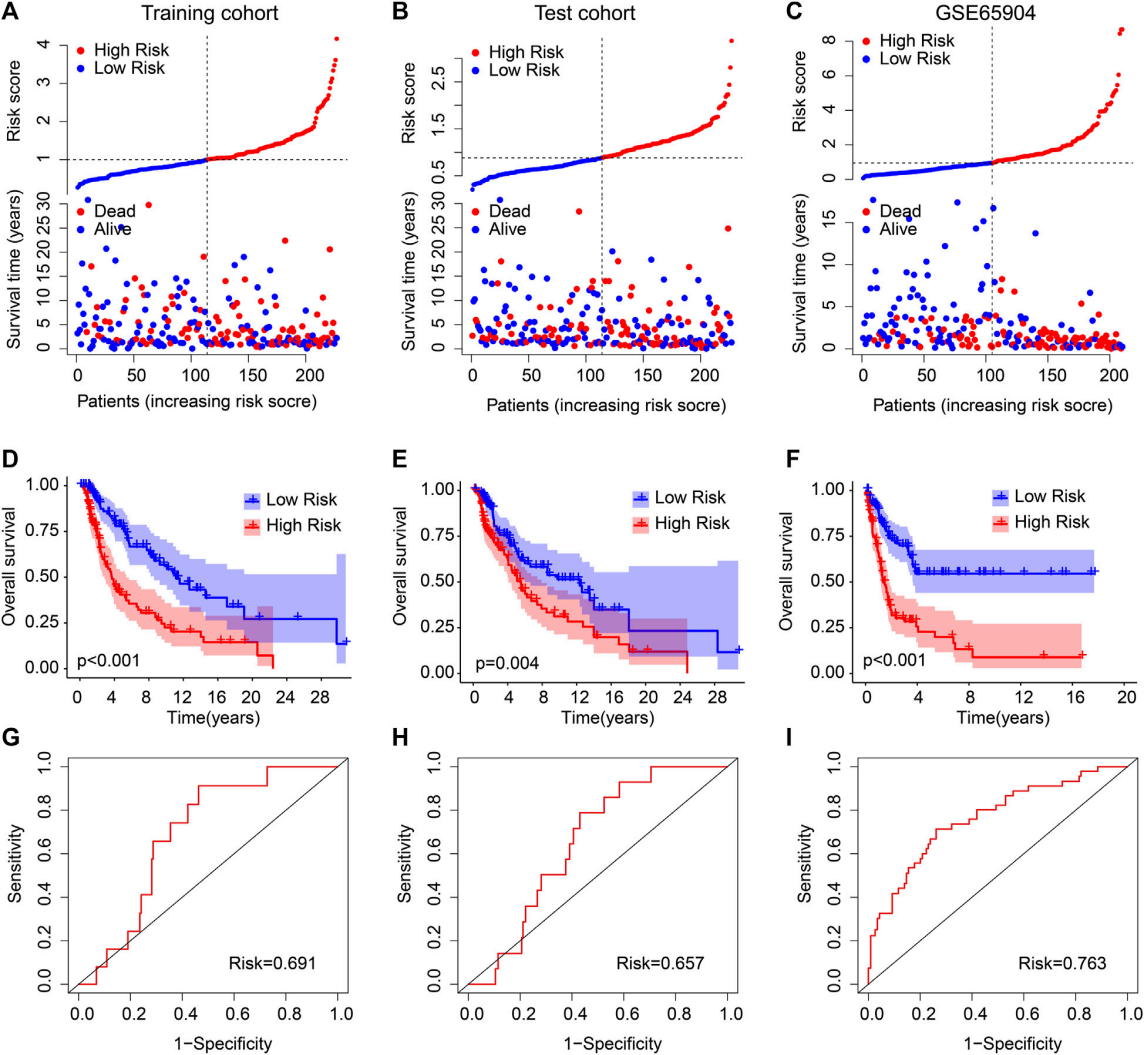
Validation of ARG prognostic signatures in CM. Risk model construction based on the ARG prognostic signatures in **(A)** training cohort, **(B)** test cohort, and **(C)** GEO cohort. The Kaplan–Meier survival curve analysis of the patients in the **(D)** training cohort, **(E)** test cohort, and **(F)** GEO cohort. The AUC of the risk model in the **(G)** training cohort, **(H)** test cohort, and **(I)** GEO cohort.

The independence and accuracy of the ARG prognostic signatures in evaluating the prognosis for CM were further investigated. CM patients were divided into a training cohort and a test cohort based on the ARG prognostic signatures. The CM patients in cohorts were ranked referring to the ARG prognostic signatures and the median risk score. The survival time was significantly correlated with the risk score revealed by the scatter dot plot (Figures 2A, B). Conversely, the contrary phenomenon was observed in the GEO cohort (Figure 2C). In line with the findings of the training and test cohorts (Figures 2D, E), the Kaplan–Meier survival curve analysis indicated that high-risk patients had a worse OS rate than the low-risk patients in the GEO cohort (Figure 2F). The AUCs of the new prognostic model were .691, .657, and .763 in the training, test, and GEO cohort, respectively (Figures 2G–I). Together, these findings demonstrate that the prediction ability of the risk model based on the ARG prognostic signatures is highly accurate and reliable.

### Correlation of clinicopathological characteristics and the ARG prognostic signatures

**FIGURE 3:**
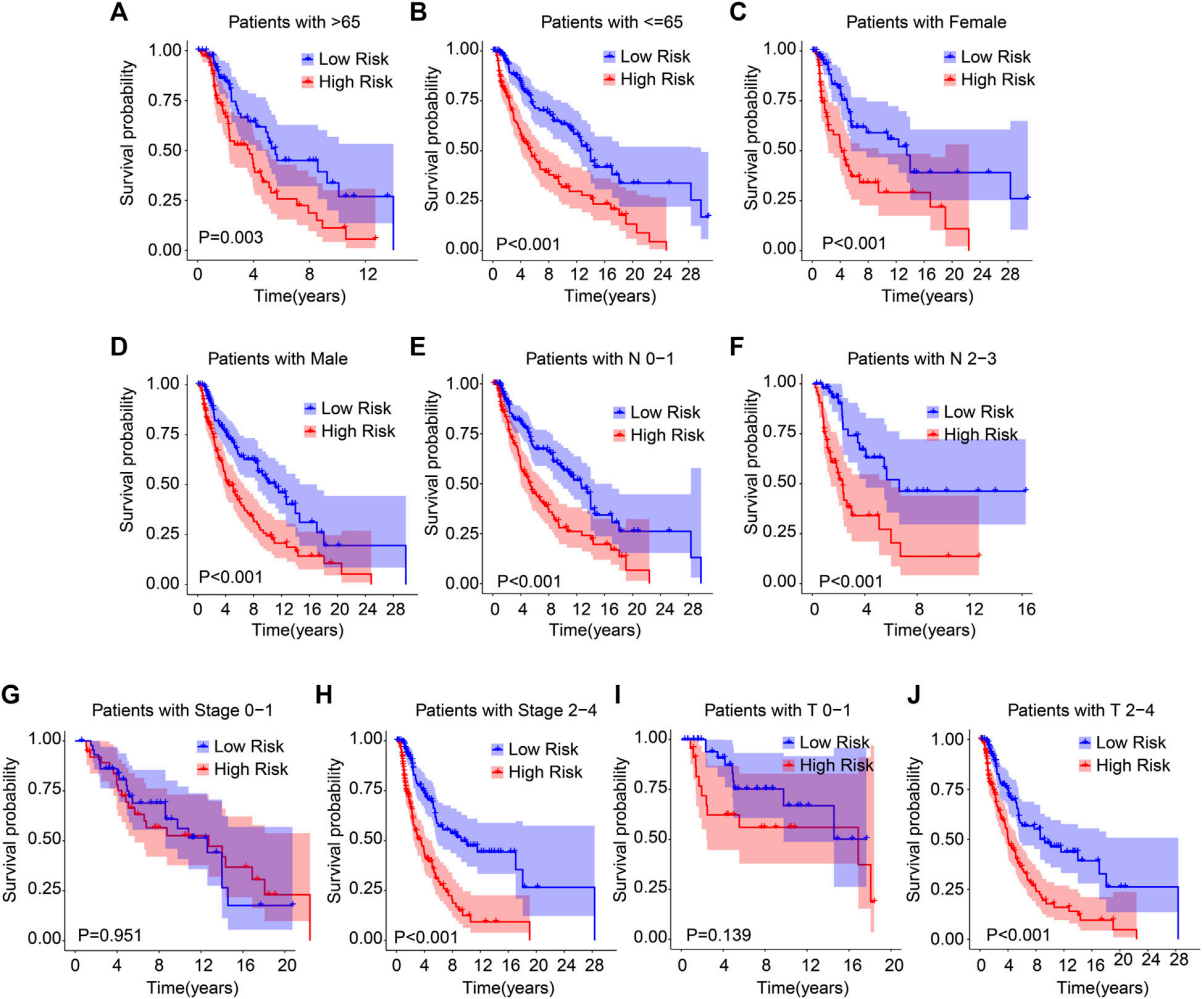
The Kaplan–Meier survival analysis of CM patients in the different clinicopathological characteristic subgroups. The Kaplan–Meier survival curve analysis shows the OS rate of patients in the low- and high-risk groups among the **(A, B)** age (age > 65 vs. age ≤ 65), **(C, D)** gender (female vs. male), **(E, F)** N (N 0–1 vs. N 2–3), **(G, H)** stage (stage 0–1 vs. stage 2–4), **(I, J)** T (T 0–1 vs. T 2–4).

Subgroup analysis was employed to further validate the prognostic roles of ARG prognostic signatures in the different important clinicopathological characteristics. Subsequently, the median of ARG prognostic signatures is used in combination with clinicopathological characteristics to classify the CM patients into the low- and high-risk groups. The results of the Kaplan–Meier survival curve analysis suggested that the patients with low-risk scores had a higher OS rate among the age >65, age ≤65, female, male, N 0–1, N 2–3, stage 2–4, and T 2–4 groups. Moreover, the OS rate of patients with low-risk scores was similar to the OS rate of patients with high-risk scores in stages 0–1 and T 0–1 (Figure 3). Collectively, above findings demonstrate that the prognostic role of the ARGs risk model could more exactly evaluate the CM prognosis relative to the clinicopathological characteristics.

## ARG prognostic signatures of CM are an independent prognosis predictor

**FIGURE 4:**
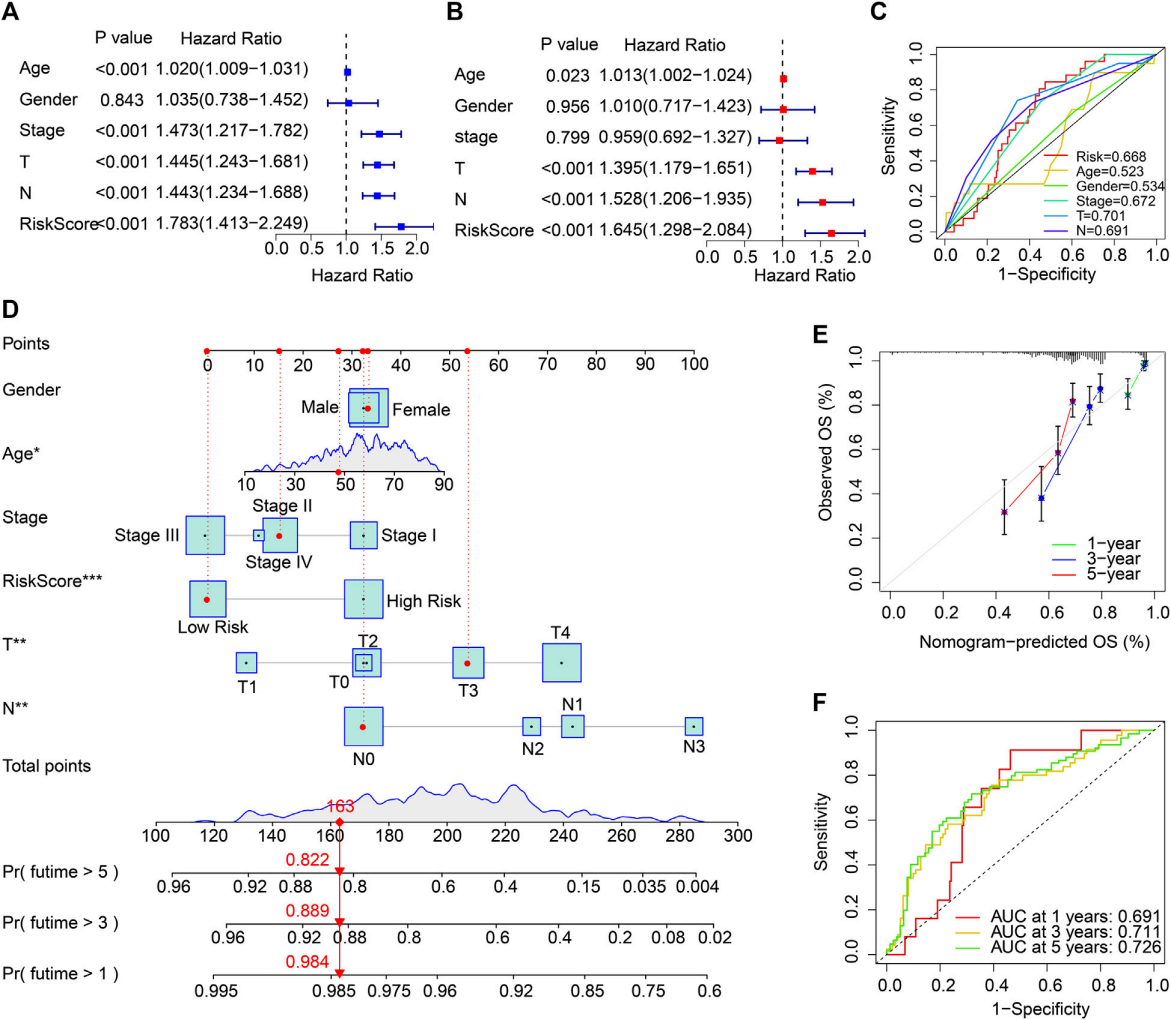
Independent ARG prognostic signature-based prognosis analysis and clinicopathological characteristics in the TCGA cohort. **(A)** The association results suggested by univariate Cox regression analyses. **(B)** Multivariate Cox regression analyses indicate that the ARG prognostic signature is an independent prognosis predictor for CM. **(C)** ROC curve displays the AUC of ARG prognostic signatures and other clinicopathological characteristics. **(D)** Nomogram of the ARG prognostic signatures and clinicopathological characteristics to predict the survival time of patients with CM. **(E)** Calibration curve analysis of the 1-, 3-, and 5-year OS rates predicted by nomogram and actual OS rates. **(F)** The AUC of the time-dependent ROC curve.

Cox regression analysis was employed to identify the independence of the ARG prognostic signatures for CM. As shown in Figure 4A, age, stage, T, N, and risk score were strongly associated with the OS rate in CM. Multivariate Cox regression analysis further suggested that age (*p* = .023), T, N, and risk score were independent prognostic predictors in CM (Figure 4B). The ROC analysis displayed that the AUC of ARG prognostic signatures was .668 (Figure 4C). Thereafter, 1-, 3-, and 5-year survival probabilities for CM patients were evaluated by a nomogram model based on the ARG prognostic signatures and other clinicopathological characteristics (Figure 4D). Satisfactory agreement with predictions was illustrated in the calibration curve result (Figure 4E). Time-dependent ROC curves for the 1-, 3-, and 5-year OS rates were plotted with AUCs of .691, .711, and .726, respectively (Figure 4F).

The same essential analyses were performed in the GEO cohort to further confirm the above results. The same Cox regression analysis results can also be seen in Supplementary Figures S1A, B. The ROC analysis showed that the AUCs of risk score, age, and gender were .763, .510, and .480, respectively (Supplementary Figures S1C). The ARG-based risk model could accurately predict the survival probability of CM patients through the nomogram and calibration curve results (Supplementary Figures S1D, E). The time-dependent ROC curve also suggested a favorable stability of the ARG prognostic power (Supplementary Figures S1F). Taken together, the risk score calculated by the prediction model based on ARGs is an independent prognosis factor for CM, and an ARG prognostic signature-based nomogram to predict the survival probability of CM is precise and feasible.

### DEGs and functional enrichment analysis

**FIGURE 5:**
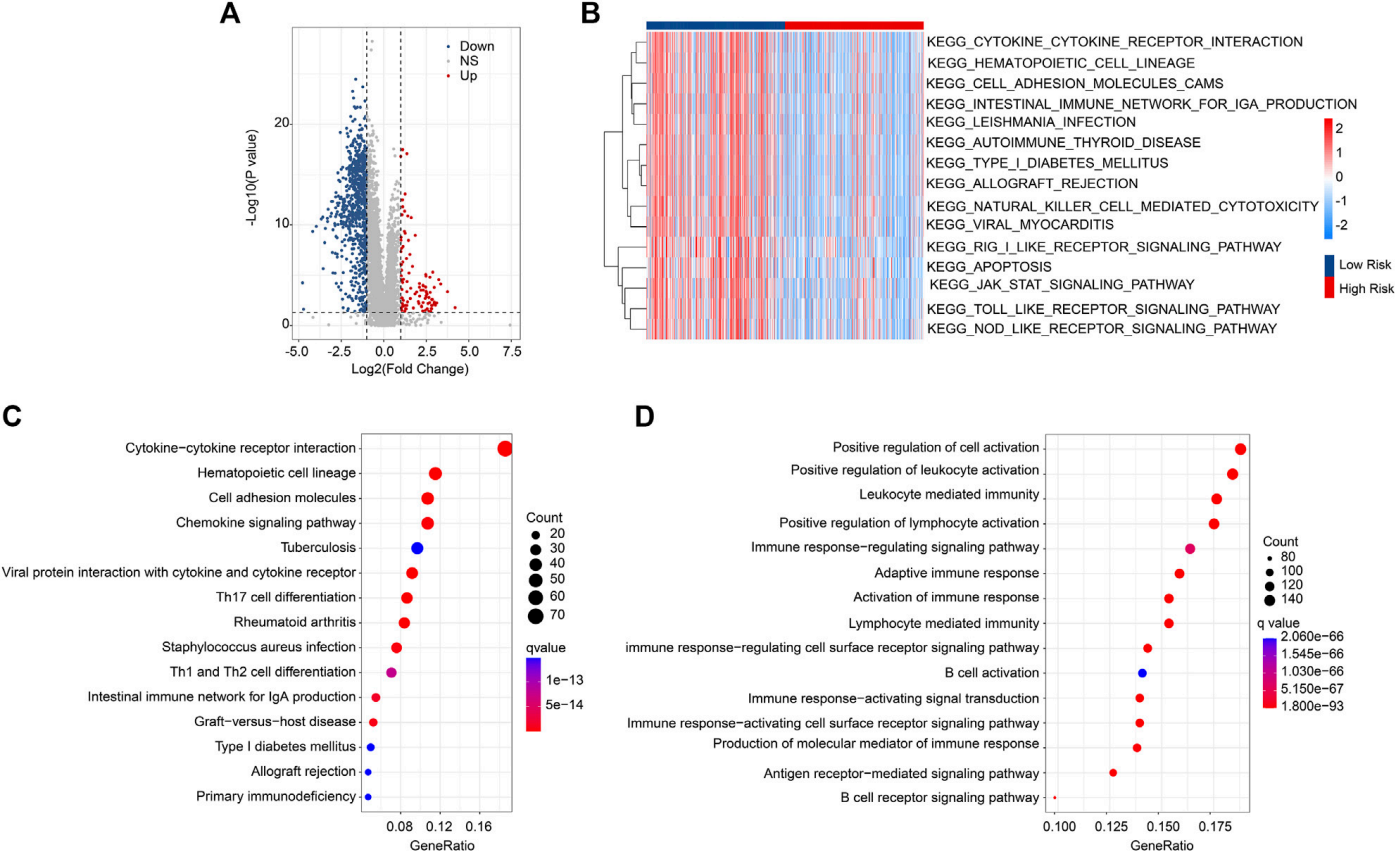
DEG Functional enrichment analysis in the different risk groups. **(A)** Volcano diagram of the significant DEGs. The threshold: |FC| ≥ 2 and *p* < .05. **(B)** GSVA analysis shows the KEGG term of CM patients in the different risk groups. **(C)** The results of KEGG enrichment analysis. **(D)** GO enrichment analysis illustrates the enriched biological process.

Enrichment analysis and GSVA revealed the underlying regulatory mechanism of DEGs in the different risk groups. The DEGs are illustrated in a volcano diagram in Figure 5A. The GSVA results indicated that signaling pathways related to the immune were significantly downregulated in the high-risk group (Figure 5B). KEGG enrichment analysis results show the cytokine-cytokine receptor interaction is dramatically enriched with DEGs (Figure 5C). GO enrichment analysis illustrated that immune-related biological process was significantly enriched, such as positive regulation of cell activation, positive regulation of leukocyte activation, and leukocyte mediated immunity (Figure 5D). These findings point to a crucial role for immune-related signaling pathways in mediating the function of ARGs in the development of CM.

### Consensus clustering and immune microenvironment landscape analysis of CM

**FIGURE 6:**
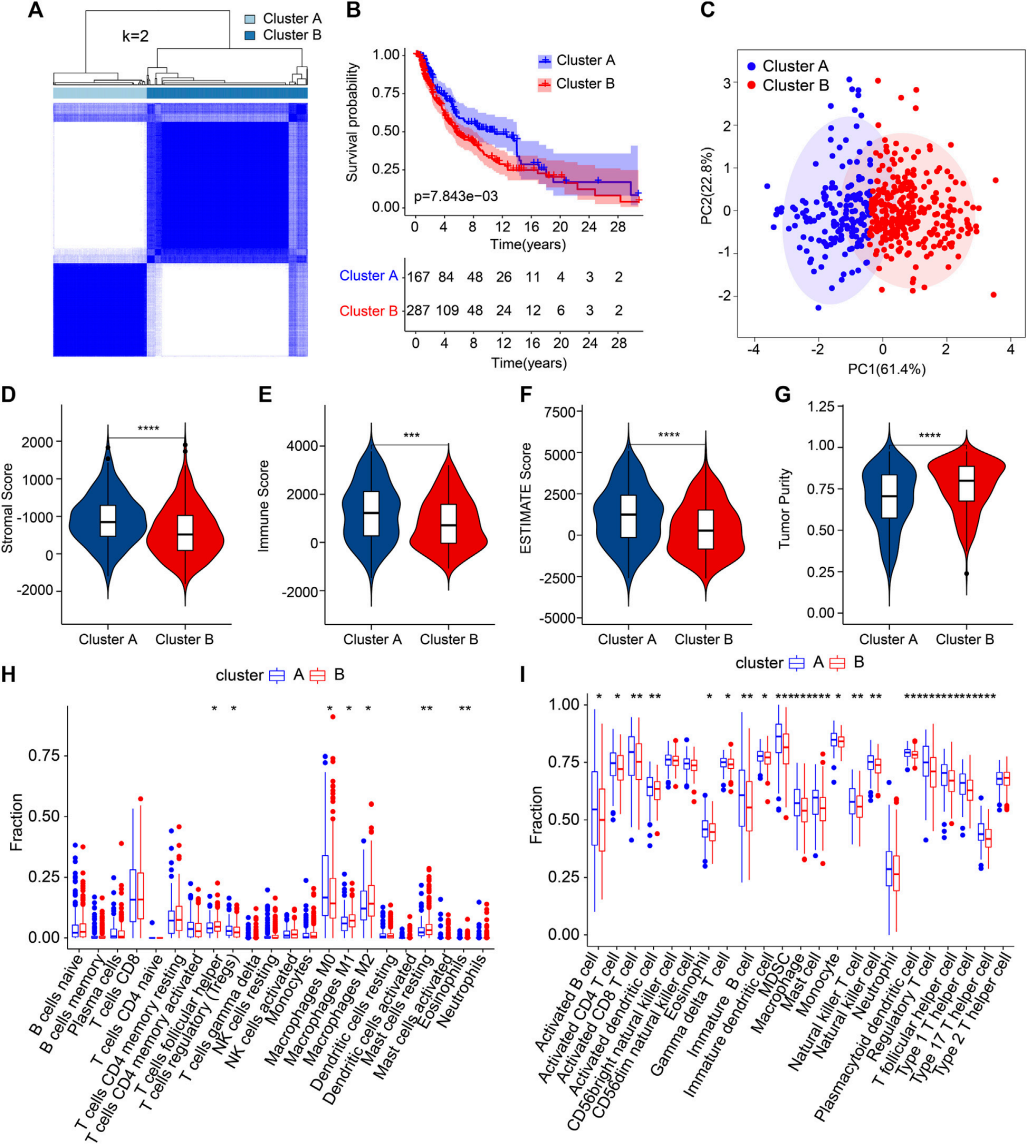
The result of consensus clustering and immune infiltration landscape analysis in different subgroups. **(A)** Consensus clustering heatmap demonstrates a suitable classification of CM samples (K = 2). **(B)** The Kaplan–Meier survival curve analysis of patients in Cluster A and Cluster B. **(C)** PCA analysis exhibits a distinction between patients in Cluster A and Cluster B according to the four prognostic ARGs. **(D–G)** The stromal, immune, and ESTIMATE scores and tumor purity results. **(H)** The fraction of 22-type immune cells in Clusters A and B. **(I)** The proportion of 23-type immune cells in Clusters A and B. *p < 0.05, **p < 0.01, ***p < 0.001, ****p < 0.0001.

The patients with CM were stratified into different subgroups depending on four prognostic ARGs by consensus clustering analysis. The consensus clustering heatmap demonstrated the classification of CM samples (K = 2), with 167 samples in Cluster A and 287 samples in Cluster B (Figure 6A). The Kaplan–Meier survival curve analyses reported that the patients in Cluster A had better OS rates (Figure 6B). It showed a clear clustering pattern according to the four prognostic ARGs on the PCA plot (Figure 6C). Next, we utilized the ESTIMATE algorithm to estimate the stromal and immune status of CM patients. Compared to the patients in Cluster B, patients in Cluster A had greater stromal, immune, and ESTIMATE scores and lower tumor purity (Figures 6D–G). The CIBERSORT algorithm suggested a higher proportion of follicular helper T cells, M0 macrophages, and regulatory T cells (Tregs) in Cluster A, whereas the proportion of M1 macrophages, resting mast cells, M2 macrophages and eosinophils were significantly higher in Cluster B (Figure 6H). ssGSEA result indicated a higher immune infiltration status of patients in Cluster A (Figure 6I). In short, these findings demonstrate that the ARGs are closely related to the prognosis and immune infiltration landscape for CM patients.

### Immune microenvironment landscape and correlation analysis of risk score

**FIGURE 7:**
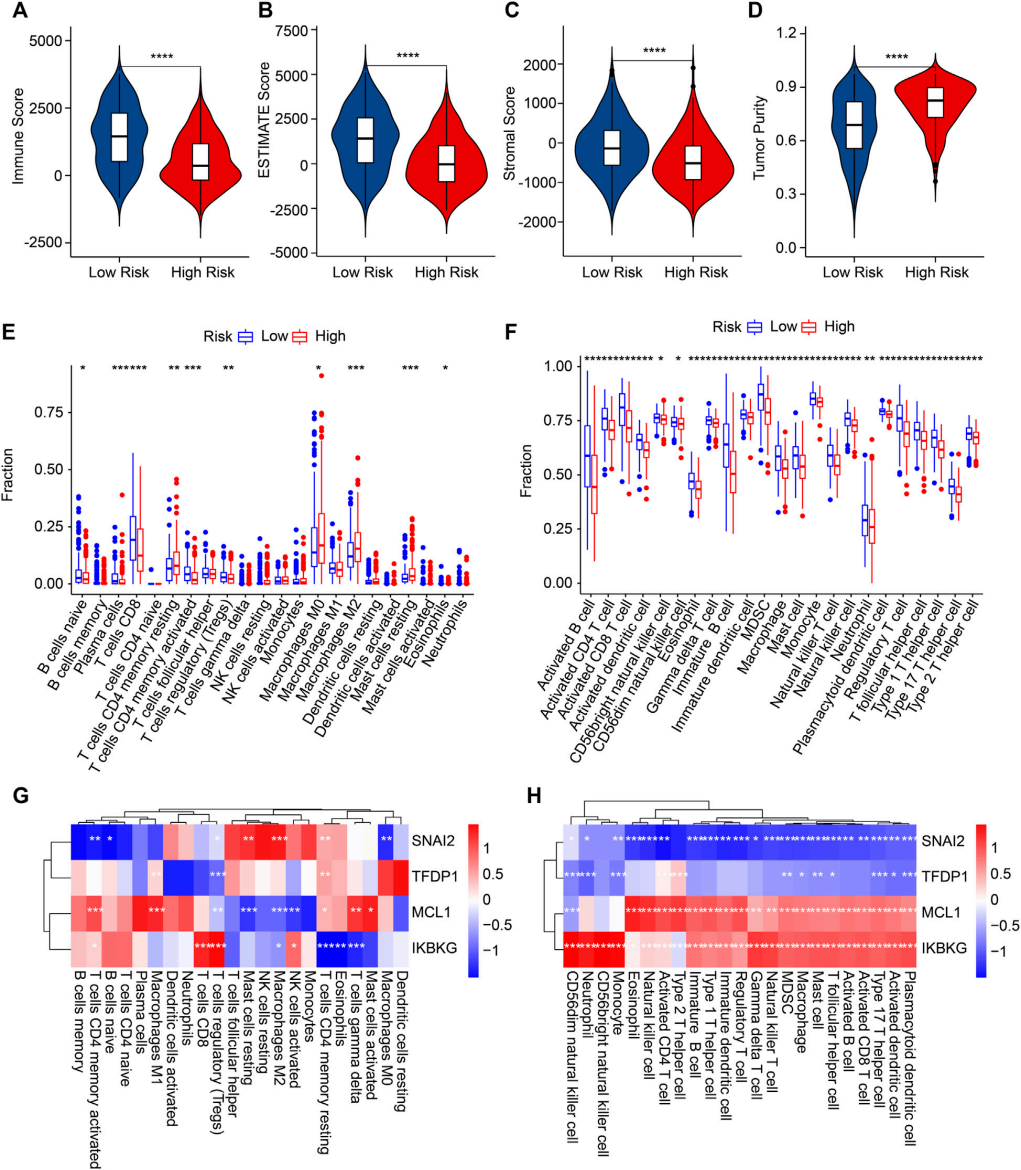
The results of immune infiltration landscape: **(A)** immune score, **(B)** ESTIMATE score, **(C)** stromal score, **(D)** tumor purity, **(E)** the proportion of 22-type cells of patients in the different risk groups, **(F)** the proportion of 23-type cells. **(G,H)** Correlation analysis of ARGs and immune cells. *p < 0.05, **p < 0.01, ***p < 0.001, ****p < 0.0001.

Multiple immune assessment algorithms were applied to better investigate the immune infiltration landscape of the patients with different risk scores. The ESTIMATE results illustrated that the low-risk score patients obtained higher immune, stromal, and ESTIMATE scores and a lower tumor purity (Figures 7A–D). The CIBERSORT result showed a higher proportion of plasma cells, CD8 T cells, naïve B cells, Tregs, CD4 memory-activated T cells, and eosinophils in the low-risk group. The proportions of CD4 memory-resting T cells, M0 macrophages, resting mast cells, and M2 macrophages were lower (Figure 7E). Compared with the high-risk score, a significantly higher percentage of 23-type immune cells were observed in the low-risk score patients, indicating a higher immune status (Figure 7F).

**FIGURE 8:**
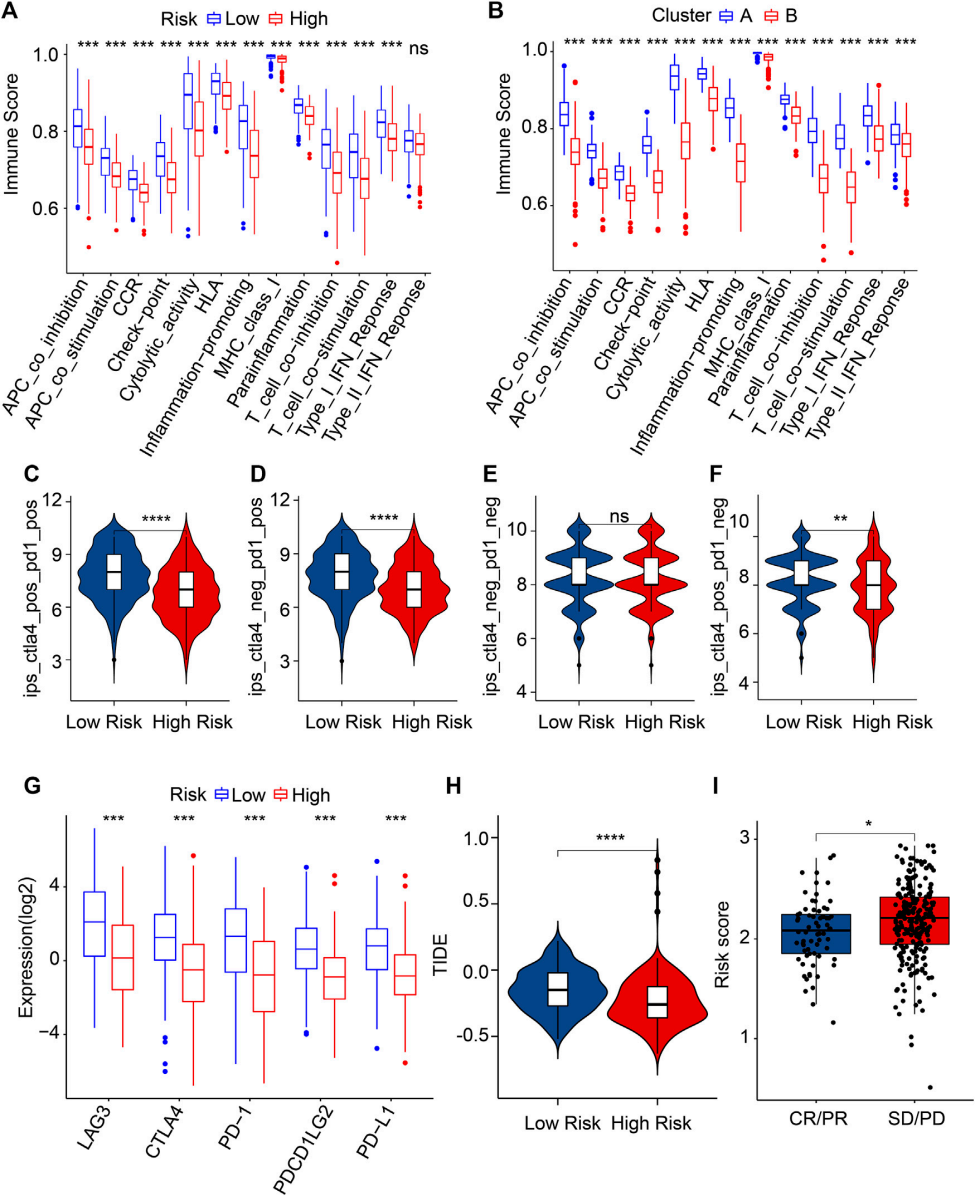
Evaluation of immune function score and immunotherapy response. **(A)** Immune function score of patients in the different risk groups. **(B)** Immune function score of patients in the clusters. **(C–F)** Immunophenoscore (IPS). **(G)** The expression of ICI in the low- and high-risk groups. The expression is transformed by log2 (expression +1). **(H)** TIDE score. **(I)** The risk score in the IMvigor210 cohort. PR, partial response; PD, progressive disease; SD, stable disease; CR, complete response. *p < 0.05, **p < 0.01, ***p < 0.001, ****p < 0.0001.

Then, the associations between four prognostic ARGs and the immune infiltration landscape were assessed by correlation analysis. As displayed in Figure 7G, a remarkable association of the four prognostic ARGs and 22-type immune cells was determined using the CIBERSORT algorithm. For example, *SNAI2* was negatively associated with M0 macrophages, naïve B cells, and CD4 memory-activated T cells but positively associated with resting mast cells, M2 macrophages, and CD4 memory-resting T cells CD4. Moreover, the correlation result illustrated a remarkable negative correlation between *SNAI2* and *TFDP1* and 23-type immune cells; *MCL1* and *IKBKG* were positively associated with the 23-type immune cells (Figure 8H). Collectively, these results demonstrate that the risk model for ARGs could reflect the immune status of CM patients.

### Risk score associated with immunotherapy response

Immunotherapy is considered the most promising treatment strategy and has attracted great attention in the clinical management of CM (Ralli et al., 2020). Given the remarkable difference in the TIME landscape of patients with CM in the different risk groups, the potential immunotherapy response of CM patients was further evaluated. The immune function score results indicated the low-risk score patients had a markedly higher immune function score than the high-risk score patients (Figure 8A). In addition, the patients in Cluster A with higher OS rates had higher immune function scores (Figure 8B). IPS results suggested that the patients with low-risk scores were sensitive to anti-*CTLA-4*, -*PD-1*, and -*CTLA-4*/*PD-1*, which suggested the potential role of immunotherapy in CM patients (Figure 8C). The result of ICI reflected that the expressions of *LAG3*, *CTLA4*, *PD-1*, *PDCD1LG2*, and *PD-L1* were higher in the patients with low-risk scores than in patients with high-risk scores (Figures 8D–F). Based on the TIDE analyses, the low-risk CM patients had a higher TIDE score (Figure 8H). As shown in Figure 8I, the risk score in the CR/PR group was markedly lower than in the SD/PD group of patients in the IMvigor210 cohort (Figure 8I). These results imply that the risk score might facilitate immunotherapy prediction for CM patients.

### The analysis of drug sensitivity

**FIGURE 9:**
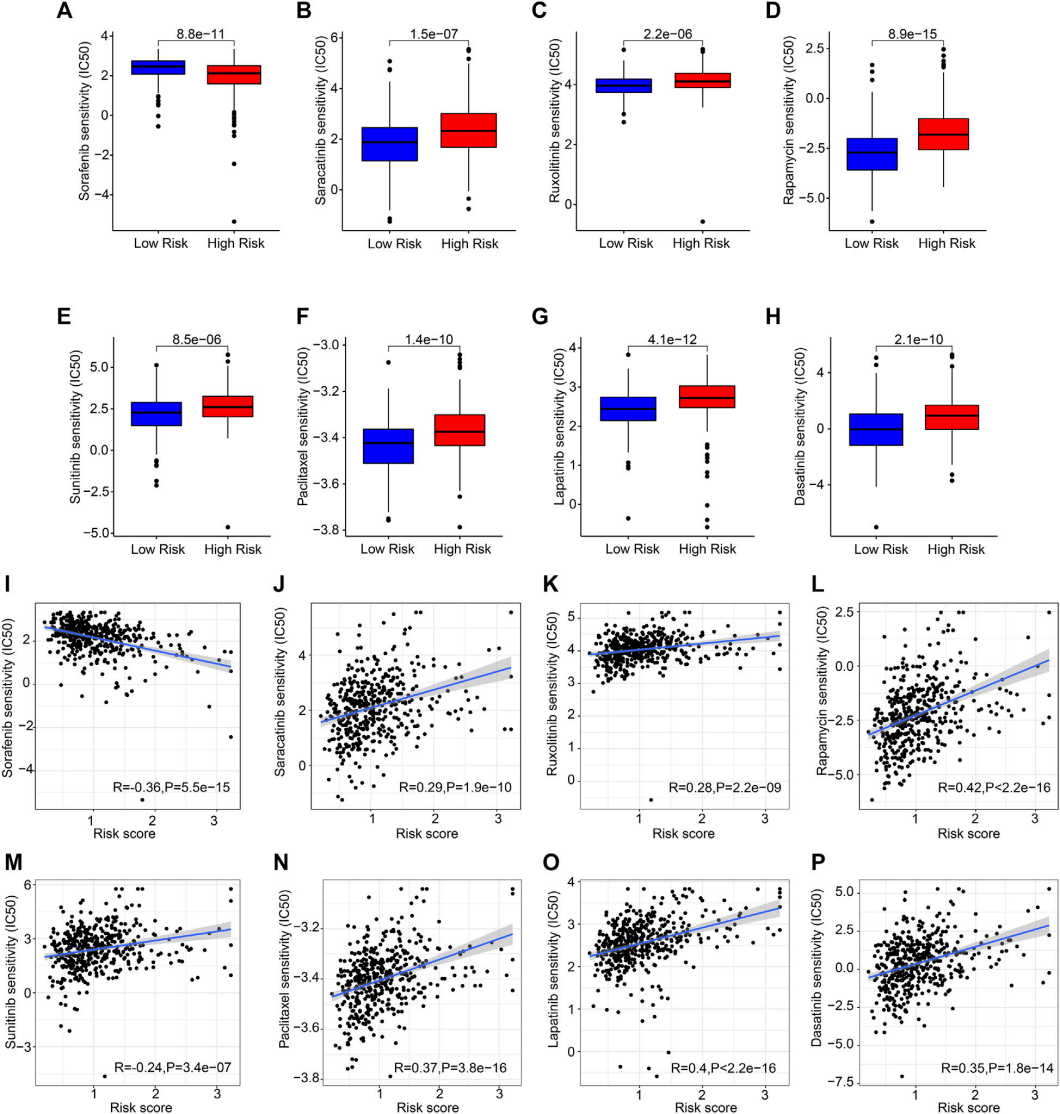
Chemotherapy response of patients in the different risk groups. **(A)** Sorafenib, **(B)** saracatinib, **(C)** ruxolitinib, **(D)** rapamycin, **(E)** sunitinib, **(F)** paclitaxel, **(G)** lapatinib, **(H)** dasatinib. **(I–P)** Correlation analysis of the risk score and drug sensitivity (IC50).

Drug targeting is another promising strategy in cancer therapy. Several antineoplastic drugs were selected to shed light on the relationship between antineoplastic drug sensitivities and risk scores. As shown in Figures 9A–H, the IC50 results revealed that the IC50 values of saracatinib, ruxolitinib, rapamycin, sunitinib, paclitaxel, lapatinib, and dasatinib were significantly higher in the high-risk group, however, the IC50 value of sorafenib was lower. The correlation analysis showed that the risk score was positively correlated with saracatinib, ruxolitinib, rapamycin, sunitinib, paclitaxel, lapatinib, and dasatinib but negatively correlated with sorafenib (Figures 9I–P). Collectively, the results presented above indicate a clear efficacy benefit for the antineoplastic drugs for patients in the different risk subgroups, giving a novel perspective for precisely targeted therapy for CM.

## Discussion

CM is a lethal malignancy with an alarming and increasing incidence rate worldwide over the past years (Song et al., 2022). It is well known that metastasis is a main factor leading to poor efficacy and prognosis of CM patients, despite the introduction of novel therapeutic approaches such as immunotherapy (Burzi et al., 2021). Thus, seeking a novel biomarker and therapeutic target is of the utmost importance. Accumulating evidence indicated that anoikis plays a vital role in tumorigenesis and development and promotes tumor invasion and metastasis (Su et al., 2013; Ness et al., 2017). Nevertheless, the characteristics of the ARGs have not yet been systematically profiled in CM. In the present study, five novel ARGs were identified as being correlated with OS. A risk model based on four ARGs was constructed to classify CM patients accurately and effectively into high- and low-risk groups. Subgroup analysis results further demonstrated that the prognostic signatures of the ARGs, which were associated with the clinicopathological characteristics, could accurately evaluate the prognosis of CM patients. The result of the enrichment analysis showed that the role of ARGs in the development of CM was mediated by immune-related signaling pathways. The analysis of the immune microenvironment landscape demonstrated the risk model based on ARGs correlated tightly with the prognosis and immune infiltration landscape of CM patients, providing new insight for CM immunotherapy. Analysis of the drug sensitivity landscape further revealed a promising new field for antineoplastic drugs for CM patients in the different risk subgroups. Overall, the current research provides a framework regarding the co-administration of targeted therapy and immunotherapy in CM treatment that may also assist in the development of individualized treatment.

A few CM prognosis models have been established, such as the hypoxia-related risk model and the cuproptosis-related model. Meanwhile, new biomarkers related to DNA and RNA molecular mechanisms have also been investigated to study the prognosis of melanoma in recent years (Riefolo et al., 2019; Jarell et al., 2022). However, the efficacy and prognosis are poor due to the metastasis of CM. In general, anoikis has previously been described essentially as a protective mechanism in tumor biology (Chen et al., 2020). Acquisition resistance to anoikis is a hallmark of cancer cells for tumor invasion and metastasis (Tsai et al., 2021). In the present article, four ARGs were selected to construct the risk model. Here, the high expressions of *SNAI2* and *TFDP1* were observed in the high-risk score patients; however, the expression of *IKBKG* and *MCL1* was lower. Snail family transcriptional repressor 2(*SNAI2*), a member of the Snail family, was regarded as an epithelial-to-mesenchymal transition-inducing transcription factor (Peng et al., 2022); (Jin et al., 2022). Extensive research has shown that *SNAI2* plays a critical role in melanocytes, adipocytes, and germ cells, contributing to cell differentiation and tumor initiation (Zhao et al., 2016). The absence of SNAI2 is associated with the level of malignancy in melanoma (Caramel et al., 2013). *TFDP1*, an important transcription factor, could coordinate with E2F proteins to promote transcription from E2F target genes (Chen et al., 2014). Reports stated that *TFDP1* also interacts with pRB and p53 to modulate the cell cycle and apoptosis. *IKBKG* is an inhibitor of kappa light polypeptide gene enhancer in B cells and is also an NF-κB essential modulator. Notably, *IKBKG* was implicated in various cancer to promote tumorigenesis. The activation of *IKBKE* could facilitate cell transformation; suppression of *IKBKE* in cancer cell lines with *IKBKE* overexpression results in cell death (Frans et al., 2017; Boisson et al., 2019). Barbie et al. (2014) have suggested that *IKBKG* was aberrantly expressed in breast carcinomas associated with NF-κB pathway activation. Myeloid cell leukemia-1 (*MCL1*), a typical anti-apoptotic protein belonging to the oncogenic BCL-2 family, was originally identified in myeloid cells (Reissig et al., 2022). *MCL1* has been found to be overexpressed in many solid tumors, and many studies highlighted the potential importance of *MCL1* as a therapeutic target (Pereira-Castro et al., 2022; Quentmeier et al., 2022). It is worth noting that demethylzeylasteral was found to evoke the apoptosis of melanoma cells by downregulating the level of *MCL1* (Zhao et al., 2017). This extensive literature has claimed that ARGs in CM substantially contribute to tumor growth and progression. The Kaplan–Meier survival curve results in the GEO cohort showed that patients with low-risk scores had a greater OS rate. Cox regression analysis further exemplified that the risk score is a clinically independent prognosis factor for CM. Collectively, the four ARG-based risk models could accurately evaluate the prognosis for CM patients.

Immune cell infiltration reflects the TIME around the tumor tissues and reportedly performs an important role in tumorigenesis and tumor progression of CM (Dong et al., 2022). A prior study implied that multiple mechanisms are involved in regulating anoikis resistance during tumor development and metastasis; the abnormal TME also helps the cancer evade anoikis (Feng et al., 2014). In this article, following the operation of the CIBERSORT algorithm, we found a significant correlation between four ARGs and 22-type immune cells. A remarkable negative correlation was found between *SNAI2*, *TFDP1*, and immune cells, whereas the opposite relationship exists with *MCL1* and *IKBKG*. T cells and M1 macrophages are regarded as the principal effectors of antitumor immunity, while immune evasion is characterized by Tregs, mast cells, and M2 macrophages (Hong et al., 2022). Overall, the findings strongly favor that the four ARG-based risk model is strongly associated with the immune infiltration landscape, reflecting the immune status of patients with CM.

Checkpoint blockade immunotherapy has emerged as a promising direction in the clinical treatment of CM (Huang and Zappasodi, 2022). Immune checkpoint inhibitors, such as *CTLA-4*, *PD-1*, *PD-L1*, and *PD-L2*, regulated the function and inhibited the antitumor immunity of activated T cells (Zhou et al., 2021). In the current study, the IPS result suggested that low-risk score patients displayed a greater response to the anti-*CTLA-4*, anti-*PD-1*, and anti-*CTLA-4*/anti-*PD-1* of CM. Incidentally, anti-*PD-1* and anti-*CTLA4* have achieved significant therapeutic effects in metastatic melanoma, which is consistent with our results. *CTLA-4* and *PD-1* are the major negative-regulatory receptors expressed on decreasing T cell antitumor responses (Patel et al., 2022). *PD-1* inhibits T cell activation and limits immune effector responses when bound by its ligands *PD-L1* and *PD-L2*. Lymphocyte activation gene 3 (*LAG3*), a key regulator of immune homeostasis, negatively regulates T cell immune responses and homeostasis, mainly by inhibiting T cell activation and proliferation (de Vos et al., 2022). Our results indicated that the low-risk score group had a higher expression level of *LAG3*, *CTLA4*, *PD-1*, *PDCD1LG2*, and *PD-L1.* Taken together, these results show that patients in the different risk subtypes respond differently to immunotherapy, indicating a fresh insight for the future individualized immunotherapy for CM.

In summary, a prognostic risk model based on ARGs was constructed and successfully divided CM patients into low- and high-risk groups in the present study. Our findings indicate that the prognostic signature established by four ARGs is a new promising model for predicting the prognosis of CM. Landscape analysis of the TIME reveals that the signaling pathways related to immunity may mediate the role of ARGs in CM. The present study demonstrates the risk impact on CM management and clinical decisions using an ARG-based risk model. However, no clinical data, such as immunochemistry and FISH test results, are available to verify this diagnostic (Gerami et al., 2012; Jarell et al., 2022). Future research will address this point. Collectively, the present study provides potential new biomarkers for the development of individualized therapeutic targets for CM.

## Data Availability

The original contributions presented in the study are included in the article/Supplementary Material; further inquiries can be directed to the corresponding authors.
