# Supplementary material for: A novel risk model based on anoikis: Predicting prognosis and immune infiltration in cutaneous melanoma
Source: Front Pharmacol. 2023 Jan 16;13:1090857. doi: 10.3389/fphar.2022.1090857 (PMC9884695; doi:10.3389/fphar.2022.1090857)
Supplement: Supplementary file 1 [file Table2.DOCX]

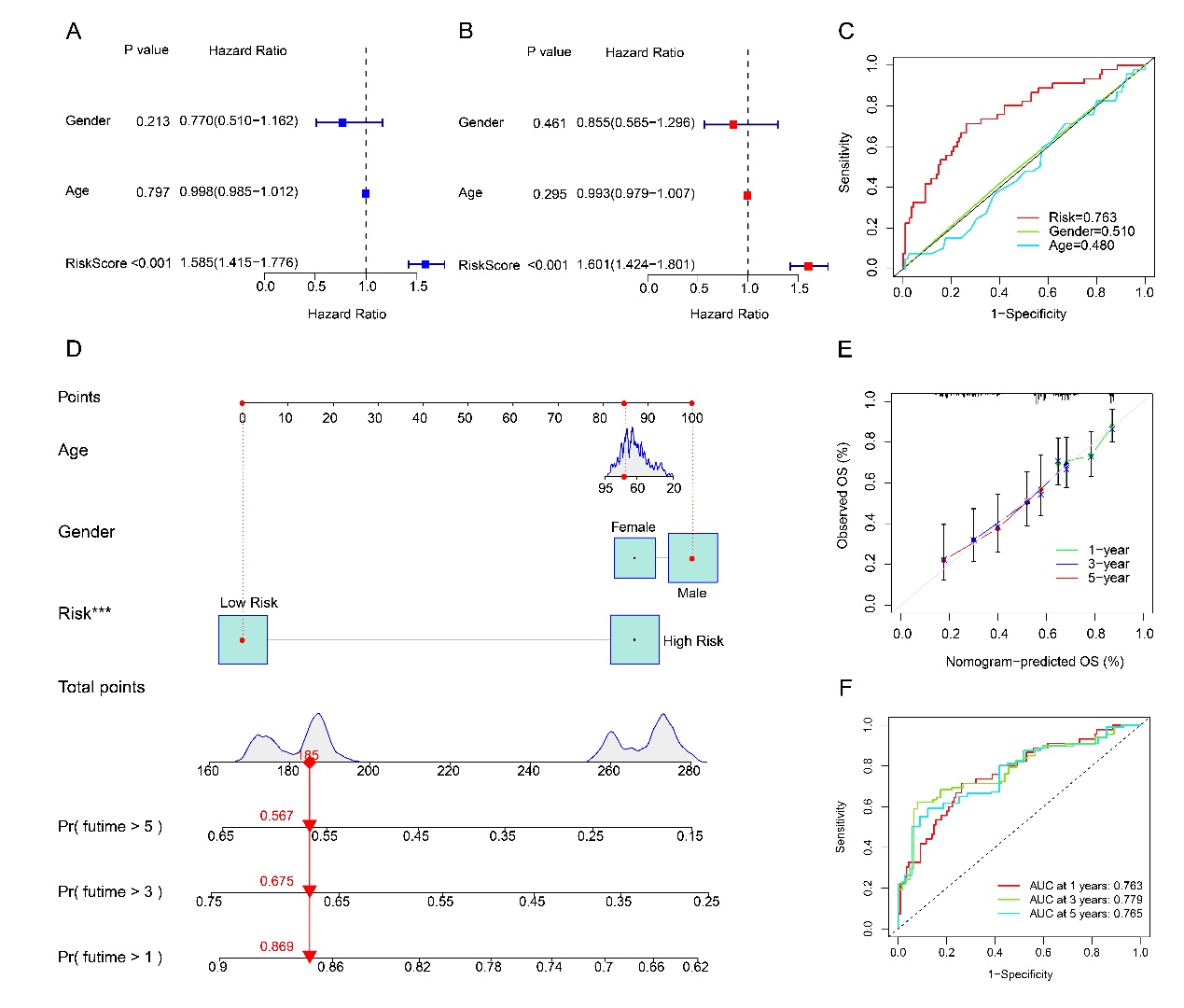


Supplement Figure 1. Independent prognosis analysis based on the ARGs prognostic signature and clinicopathological characteristics in GEO cohort. （A, B） Univariate and Multivariate Cox regression analyses indicates that the ARGs prognostic signature is an independent prognosis predictor for CM. （C） ROC curve shows the AUC of ARGs prognostic signature and other clinicopathological characteristics. （D） Nomogram construction of the ARGs prognostic signature and clinicopathological characteristics to predict the 1-, 3-, and 5-year’s survival time of patients with CM. （E） Calibration curve analysis shows the consistency of the 1-, 3-, and years OS rate predicted by nomogram and actual OS rate. （F） Time-dependent ROC curve shows the AUC at 1-, 3-, and 5-years.
